# Tumor xenograft modeling identifies an association between TCF4 loss and breast cancer chemoresistance

**DOI:** 10.1242/dmm.032292

**Published:** 2018-05-18

**Authors:** Gorka Ruiz de Garibay, Francesca Mateo, Agostina Stradella, Rafael Valdés-Mas, Luis Palomero, Jordi Serra-Musach, Diana A. Puente, Ander Díaz-Navarro, Gardenia Vargas-Parra, Eva Tornero, Idoia Morilla, Lourdes Farré, María Martinez-Iniesta, Carmen Herranz, Emmet McCormack, August Vidal, Anna Petit, Teresa Soler, Conxi Lázaro, Xose S. Puente, Alberto Villanueva, Miguel Angel Pujana

**Affiliations:** 1 Breast Cancer and Systems Biology Laboratory, Program Against Cancer Therapeutic Resistance (ProCURE), Catalan Institute of Oncology (ICO), Oncobell, Bellvitge Institute for Biomedical Research (IDIBELL), L'Hospitalet del Llobregat, Barcelona 08908, Catalonia, Spain; 2 Department of Medical Oncology, ICO, Oncobell, IDIBELL, L'Hospitalet del Llobregat, Barcelona 08908, Catalonia, Spain; 3 Department of Biochemistry and Molecular Biology, Instituto Universitario de Oncología del Principado de Asturias, Universidad de Oviedo, Oviedo 33006, Spain; 4 Hereditary Cancer Programme, ICO, Oncobell, IDIBELL, L'Hospitalet del Llobregat, Barcelona 08908, Catalonia, Spain; 5 Chemoresistance and Predictive Factors Laboratory, ProCURE, ICO, Oncobell, IDIBELL, L'Hospitalet del Llobregat, Barcelona 08908, Catalonia, Spain; 6 Departments of Clinical Science and Internal Medicine, Haematology Section, Haukeland University Hospital, and Centre for Cancer Biomarkers CCBIO, Department of Clinical Science, University of Bergen, Bergen 5021, Norway; 7 Department of Pathology, University Hospital of Bellvitge, Oncobell, IDIBELL, L'Hospitalet del Llobregat, Barcelona 08908, Catalonia, Spain; 8 Biomedical Research Networking Centre of Cancer, CIBERONC, Spain; 9 Xenopat S.L., Business Bioincubator, Bellvitge Health Science Campus, L'Hospitalet del Llobregat, Barcelona 08908, Catalonia, Spain

**Keywords:** Breast cancer, Chemotherapy, Resistance, TCF4, Xenograft, Transcription factor, Patient-derived xenograft

## Abstract

Understanding the mechanisms of cancer therapeutic resistance is fundamental to improving cancer care. There is clear benefit from chemotherapy in different breast cancer settings; however, knowledge of the mutations and genes that mediate resistance is incomplete. In this study, by modeling chemoresistance in patient-derived xenografts (PDXs), we show that adaptation to therapy is genetically complex and identify that loss of transcription factor 4 (TCF4; also known as ITF2) is associated with this process. A triple-negative *BRCA1*-mutated PDX was used to study the genetics of chemoresistance. The PDX was treated in parallel with four chemotherapies for five iterative cycles. Exome sequencing identified few genes with *de novo* or enriched mutations in common among the different therapies, whereas many common depleted mutations/genes were observed. Analysis of somatic mutations from The Cancer Genome Atlas (TCGA) supported the prognostic relevance of the identified genes. A mutation in *TCF4* was found *de novo* in all treatments, and analysis of drug sensitivity profiles across cancer cell lines supported the link to chemoresistance. Loss of TCF4 conferred chemoresistance in breast cancer cell models, possibly by altering cell cycle regulation. Targeted sequencing in chemoresistant tumors identified an intronic variant of *TCF4* that may represent an expression quantitative trait locus associated with relapse outcome in TCGA. Immunohistochemical studies suggest a common loss of nuclear TCF4 expression post-chemotherapy. Together, these results from tumor xenograft modeling depict a link between altered TCF4 expression and breast cancer chemoresistance.

## Abstract (precis)

**Summary:** By modeling chemoresistance in patient-derived breast cancer xenografts, this study shows that adaptation to therapy is genetically complex and that loss of transcription factor 4 (TCF4) is associated with this process.

## INTRODUCTION

Triple-negative breast cancer (TNBC), defined by the absence (or relative low expression) of the estrogen and progesterone receptors (ER and PR, respectively) and of human epidermal growth factor receptor 2 (HER2), accounts for 15-20% of all breast cancer cases (Foulkes et al., 2010). The lack of the defined molecular markers means that the standard targeted treatments currently available for other settings (i.e. endocrine-based or anti-HER2 therapy) are inapplicable. Conventional chemotherapy – CMF (cyclophosphamide, methotrexate and fluorouracil) and/or anthracycline- and taxane-containing regimens – is the basis of TNBC treatment according to the majority of national and international guidelines (Coates et al., 2015). These therapies typically give response rates of 30-70%, but they are often not durable, with a time to progression of 6-10 months (von Minckwitz et al., 2012). In this context, resistance may arise through a number of different mechanisms (O'Reilly et al., 2015) and tumor cell heterogeneity coupled with high mutation rates may be a key factor contributing to a rapid selection of drug-resistant clones (de Bruin et al., 2013).

Given the clinical impact of therapeutic resistance, there is renewed interest in platinum-based drugs, either as a single agent or in combination therapy. This interest is principally led by the high frequency of germline and/or somatic alterations in the *BRCA1* gene in TNBC tumors (Silver et al., 2010). However, addition of platinums in therapy increases toxicity and, most importantly, there is no conclusive evidence that these agents improve disease-free and overall survival (La Belle et al., 2017). Based on the same idea of specific vulnerabilities, alteration of DNA repair signaling is also being assessed as a therapeutic approach using, for example, poly-ADP ribose polymerase (PARP) inhibitors (Lord and Ashworth, 2013; La Belle et al., 2017). Nevertheless, chemotherapy is generally applied empirically, with no validated test or biomarker to reliably predict response to or benefit from a particular regime (Caponigro and Sellers, 2011). In this scenario, PDXs have proven to be an effective approach for the assessment of novel therapeutic approaches and are widely used in preclinical drug development (Byrne et al., 2017). PDXs provide the opportunity to place the same patient tumor under a battery of therapeutic regimens and, thus, identify useful vulnerabilities. At the same time, PDXs facilitate the study of human tumor-specific characteristics. In this study, PDX-based modeling of breast cancer chemoresistance leads to the identification of loss of TCF4 as being associated with this phenotype.

## RESULTS

### Adaptation to chemotherapy

**Fig. 1.**
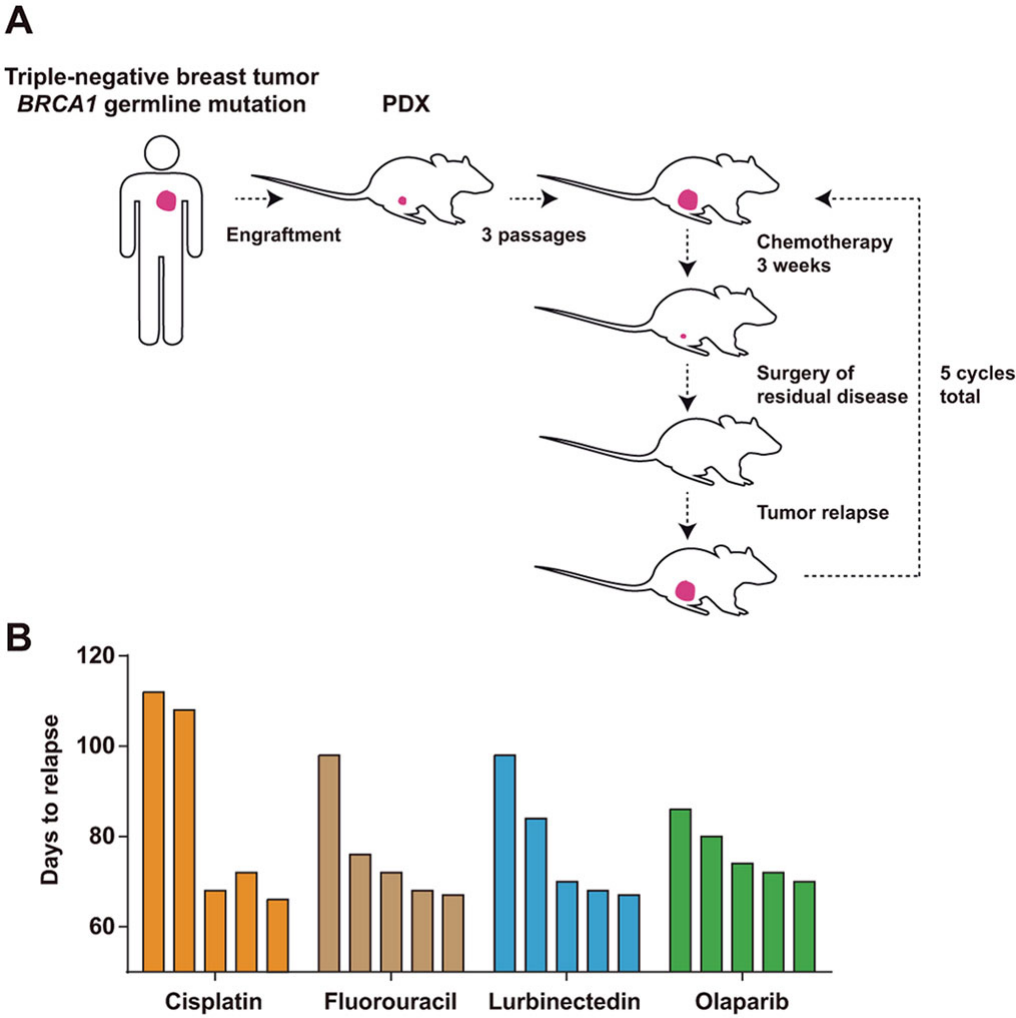
***In vivo* modeling of adaptation to chemotherapy.** (A) Strategy for the generation of *in vivo* adapted PDXs. (B) Graphs showing the observed days to relapse of the PDXs after each cycle of chemotherapy (initial assessment and four additional cycles).

A breast tumor from a 33-year-old woman carrying a pathological germline *BRCA1* mutation (c.302-1G>A) was obtained at surgery and subsequently engrafted in the mammary fat pad of female athymic mice. The patient had received four cycles of TAC (docetaxel, doxorubicin and cyclophosphamide) regimen at the time of surgery, but with no substantial clinical or pathological responses. The PDX was established for three passages before chemoresistance modeling was initiated. Next, replicates of the PDX were treated in parallel with cisplatin (a platinum-containing drug), fluorouracil (an anti-metabolite and irreversible inhibitor of thymidylate synthase), lurbinectedin (a DNA minor-groove binder) or olaparib (a PARP inhibitor). Five treatment cycles were applied, consisting of approximately 3 weeks of drug administration followed by surgery for tumor removal (Fig. 1A). Recapitulating what is commonly observed in the emergence of therapeutic resistance, the required time to relapse (defined as tumors with a volume of 500-750 mm^3^) fell in parallel with the number of treatment cycles (Fig. 1B). This trend was observed in the four drug settings, indicating that the tumors were adapted to chemotherapy. None of the treated PDXs showed reversion to wild-type status of the germline *BRCA1* mutation (Fig. S1). Thus, *in vivo* adaptation to the chemotherapies could be partially mediated by the acquisition of additional genetic alterations.

### Genetic heterogeneity linked to chemoresistance

To investigate the genetics of chemoresistance, exome sequences were obtained at an average coverage >80× for each of the four adapted chemotherapeutic settings, the parental PDX and germline samples. In total, 187 mutations were identified relative to the germline genome of the patient: six frameshift, seven nonsense, one affecting a consensus splice site and 173 missense. Sixty-four mutations were considered *de novo* since they were absent from the parental PDX at the defined exome coverage (Table S1). Forty-three mutations were completely depleted in at least one of the adapted models (Table S2), and 80 mutations were present in both the adapted and parental settings relative to the germline. Among the latter, 22 and 19 were significantly enriched (Table S3) and depleted (Table S2), respectively, in at least one of the chemoresistance models. To assess the validity of mutation calls, 21 changes were selected for Sanger-based sequence analysis; these included six frameshift and 15 point mutations (six nonsense, eight missense and one splice site). Only two of the predicted mutations, including a frameshift mutation, were not validated (Table S4). To further assess the validity of somatic mutations and precisely determine their frequencies, 20 of these changes were analyzed by deep-targeted sequencing in seven DNA samples from the same study case: from the germline, primary tumor, parental xenograft and each of the resistant xenograft models (mean coverage 8223-fold). This analysis corroborated the somatic acquisition of the mutations and expanded the identification of three of them with >10% frequency in the olaparib-resistant xenograft (Table S5). The fact that these three mutations were not initially identified in the sample used for exome and Sanger sequencing, but detected in a different sample of the same xenograft, suggests that topologically isolated sub-clones might exist in this case; however, analysis of tumor clonality was hampered by the fact that estimation of copy number alterations using the xenograft data was unreliable.

**Fig. 2.**
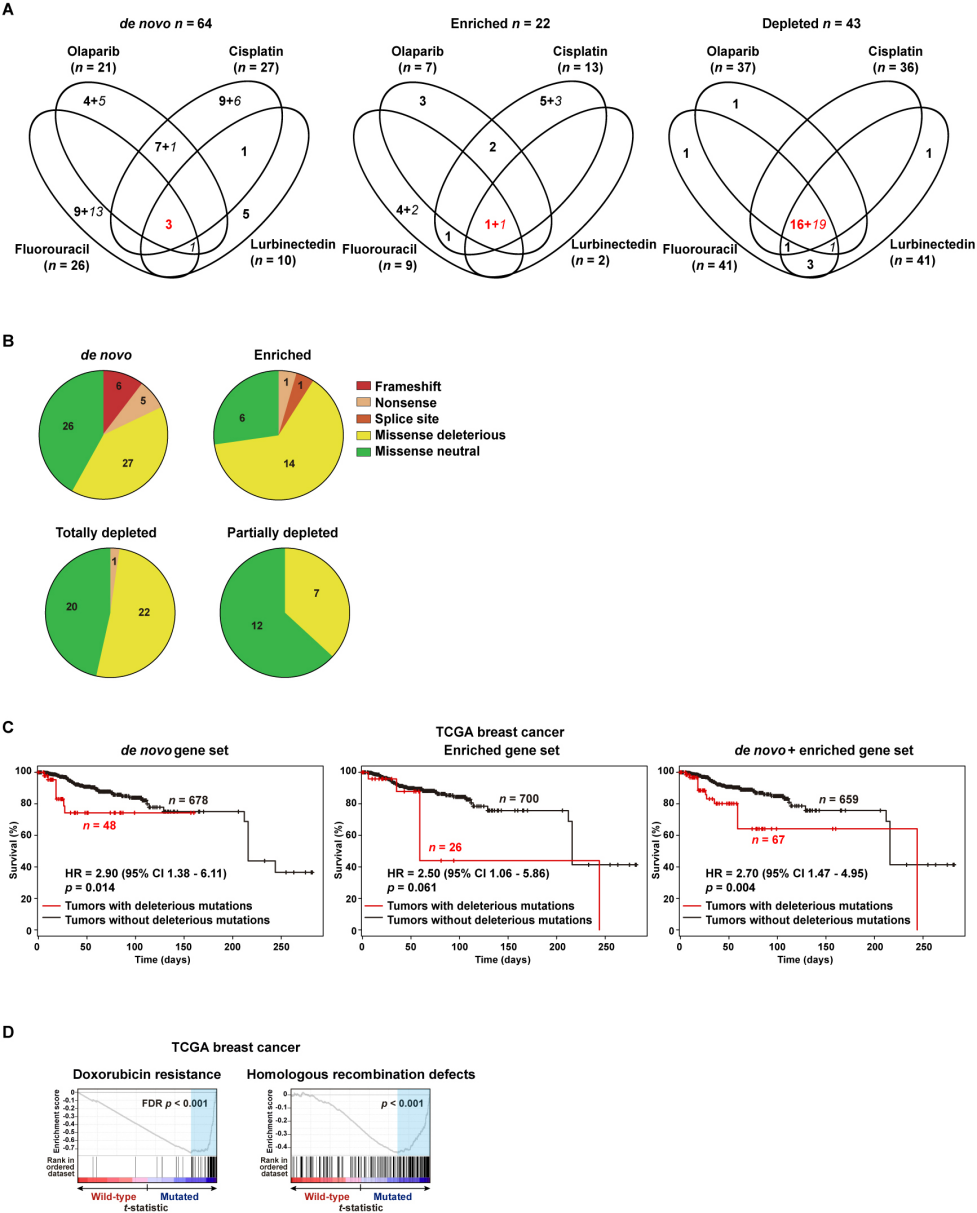
**Genes with mutations linked to adaptation to chemotherapies.** (A) Venn diagrams showing the number of identified genes with mutations (*de novo*, significantly enriched or depleted) across the four chemotherapeutic settings. Italic font marks the number of genes with non-deleterious mutations and red font indicates the number of genes identified in common. (B) Pie chart showing types of mutations and the number identified in each setting. (C) Kaplan–Meier curves showing survival rates over time for TCGA patients stratified according to the presence or absence of deleterious mutations in the defined gene sets. The HR estimations, 95% CIs and log-rank test *P*-values are shown. (D) GSEA results for the rank of differential gene expression (based on *t*-statistic) between TCGA wild-type and mutated (deleterious mutations/genes as defined in the PDXs) patients. Left panel shows results for a significant C2-curated GSEA, which defines resistance to doxorubicin. Right panel shows the result of the analysis of a set previously associated with homologous recombination defects and chemoresistance.

Next, the exome-based mutations and target genes identified across the adapted models were compared. Intriguingly, 55 of 64 *de novo* mutations were found to be specific to a single chemotherapeutic setting. Similarly, 17 of 22 enriched mutations were specific to a single setting. By contrast, 36 of the 43 completely or partially depleted mutations (note that a given variant can be classified as completely or partially depleted in different settings) were identified in common among the four adapted models (Fig. 2A; includes functional predictions, see subsequent section). These data suggest that diverse mutations and genes can potentially mediate adaptation to chemotherapy.

### Functional and prognostic relevance of deleterious mutations

Deleterious missense mutations were defined as being similarly predicted by both the SIFT (Sim et al., 2012) and Polyphen-2 (Adzhubei et al., 2013) algorithms. Including frameshift, nonsense and splice site mutations, ∼60% and ∼70% of the *de novo* and enriched mutations, respectively, were considered deleterious. By contrast, a lower percentage of the completely and partially depleted missense mutations were considered deleterious (∼50% and ∼40%, respectively; Fig. 2B). In fact, the proportion of predicted deleterious mutations in the set of enriched changes was found to be significantly higher than in the set of partially depleted changes (*Z*-score=2.08, *P*=0.037), further reinforcing the link between enriched somatic mutations and adaptation to chemotherapies.

Next, we assessed the biological significance of the identified mutations by examining gene annotations corresponding to Kyoto Encyclopedia of Genes and Genomes (KEGG) pathways (Kanehisa et al., 2017). While the set of depleted mutations did not show any enrichment, the set of acquired (*de novo* plus enriched) mutations was found to be over-represented [false discovery rate (FDR) <5%] in genes belonging to the non-homologous end-joining (NHEJ) pathway: these genes and their corresponding mutations were *DNTT* (p.Val237Leu) and *XRCC6* (p.Val482Cysfs*16) (Table S4). This observation is consistent with the known role of the NHEJ pathway (as an error-prone process) in mediating chemoresistance (Jeggo and Löbrich, 2007).

Following on from the indication of genetic heterogeneity in adaptation, the prognostic significance of the mutational signatures identified above was evaluated using somatic data from TCGA (Cancer Genome Atlas Network, 2012). Thus, TCGA cases were classified in subsets depending on whether their tumors carried deleterious mutations in any of the genes from the three sets described above (i.e. *de novo*, enriched or depleted mutations/genes) or were wild-type for all of these genes. In this classification, deleterious mutations were defined as being a frameshift, nonsense, affecting a splice site, or missense predicted to be deleterious by both SIFT and Polyphen-2 (Table S6). Next, the prognosis of each TCGA subset was assessed relative to wild-type cases. Thus, patients whose tumors carried a deleterious mutation corresponding to the *de novo* or enriched PDX-derived gene sets tended to have poorer overall survival, and the combined analysis was significant: hazard ratio (HR)=2.70, 95% confidence interval (CI) 1.47-4.95, *P*=0.004 (Fig. 2C). By contrast, those patients with somatic missense mutations predicted to be neutral in the *de novo* and enriched gene sets did not show a significant difference in prognosis: HR=1.73, 95% CI 0.89-3.34, *P*=0.12. The number of events limited the scope for analysis by cancer subtypes.

Next, the genome-wide expression profiles of the mutated TCGA subset as defined above were analyzed for their association with previously proven signatures of chemoresistance. Thus, the mutated TCGA tumors showed a significant positive correlation (FDR<5%) with a signature of resistance to doxorubicin (Kang et al., 2004) (Fig. 2D, left panel). In addition, specific analysis of a signature of homologous recombination defects and chemoresistance (Peng et al., 2014) also showed a positive correlation (Fig. 2D, right panel). Therefore, the proportions of deleterious mutations and their functional (including co-expression) and prognostic associations indicate that a relevant portion of the identified genetic alterations mediate adaptation to chemotherapies.

### TCF4 loss mediates chemoresistance

**Fig. 3.**
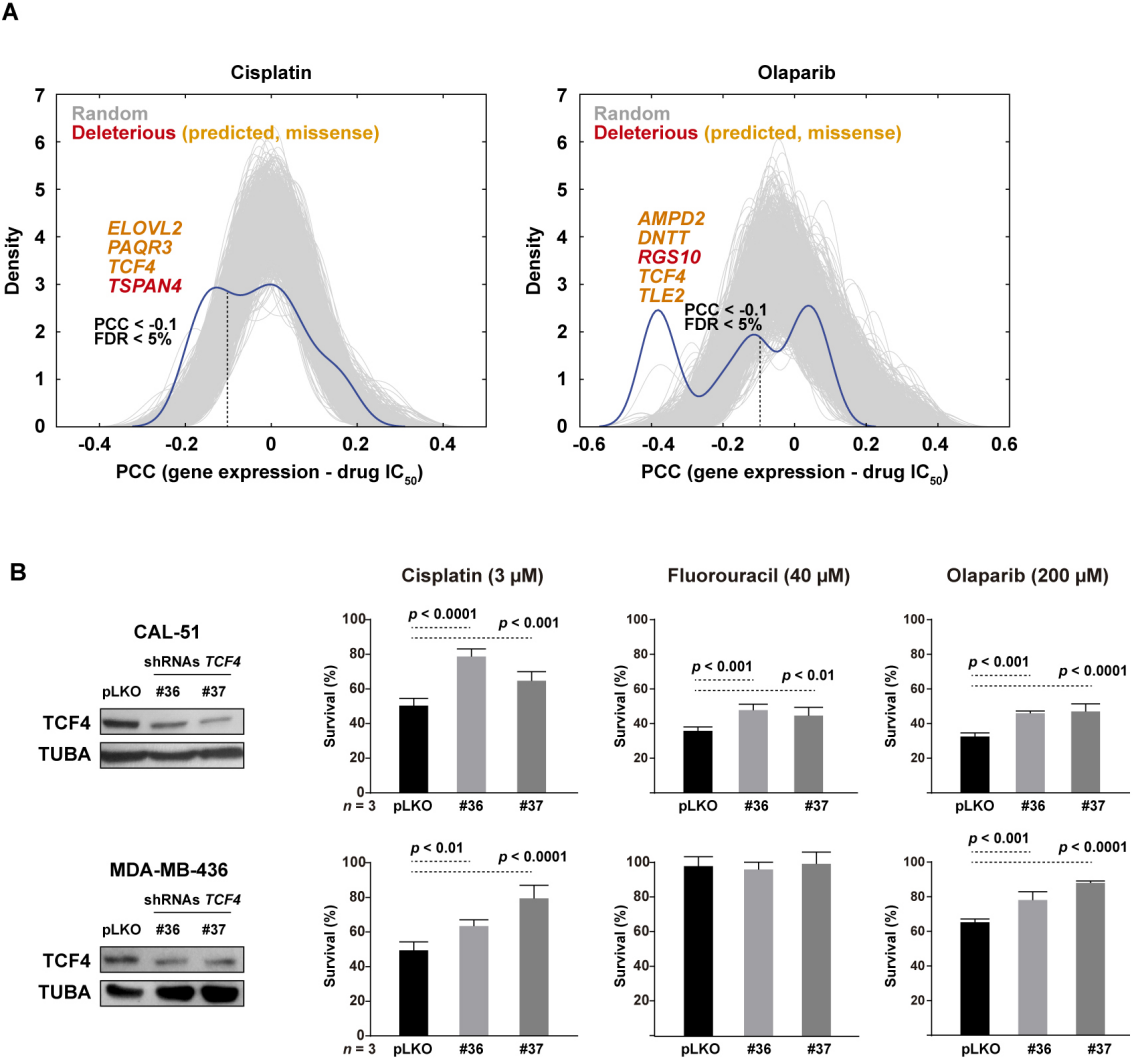
***TCF4* mediates chemoresistance.** (A) Graphs showing the distribution of correlations (PCCs) between the expression of genes with deleterious mutations (including predicted missense; genes marked in orange) and the IC_50_ values for cisplatin (left panel) and olaparib (right panel) across cell lines of the Genomics of Drug Sensitivity dataset. The results for 1000 random sets of genes of equivalent size are shown as gray curves. The threshold of significant (FDR <5%) negative PCCs is marked in both graphs by a vertical dashed line. (B) shRNA-mediated depletion of *TCF4* expression confers resistance to cisplatin, olaparib and fluorouracil in CAL-51 cells (top panels), and to cisplatin and olaparib in MDA-MB-436 cells (bottom panels; this cell model shows resistance to fluorouracil at basal conditions). The drug exposures lasted 72 h at the depicted concentrations. ANOVA *P-*values corrected by multiple testing are shown.

To further evaluate the identified genes, we assessed their expression correlation with the half maximal inhibitory concentration (IC_50_) profiles to cisplatin and olaparib across hundreds of cancer cell lines (Garnett et al., 2012). Compared to 1000 randomly chosen gene sets of equivalent size, the expression of genes with deleterious mutations in our study was found to be enriched in negative correlations with drug responses (Mann–Whitney test *P*-values<0.001; Fig. 3A); that is, low expression of genes identified with deleterious mutations in PDX-based modeling is frequently associated with higher IC_50_ values for cisplatin and olaparib in cancer cell lines, which reinforces their link to adaptation. Among the identified genes, *TCF4* showed the lowest negative correlation with both IC_50_ drug profiles [Pearson's correlation coefficients (PCCs)=−0.16 and −0.38, respectively, *P*-values<0.001]. The cancer cell line dataset did not include therapeutic results for fluorouracil and lurbinectedin. The *TCF4* gene was identified with a common *de novo* mutation in all of our four therapeutic assays: c.893C>A (NM_003199) leading to proline to histidine at position 298 (Fig. S2), which is within a potential TPP phosphorylation (T297) motif.

**Fig. 4.**
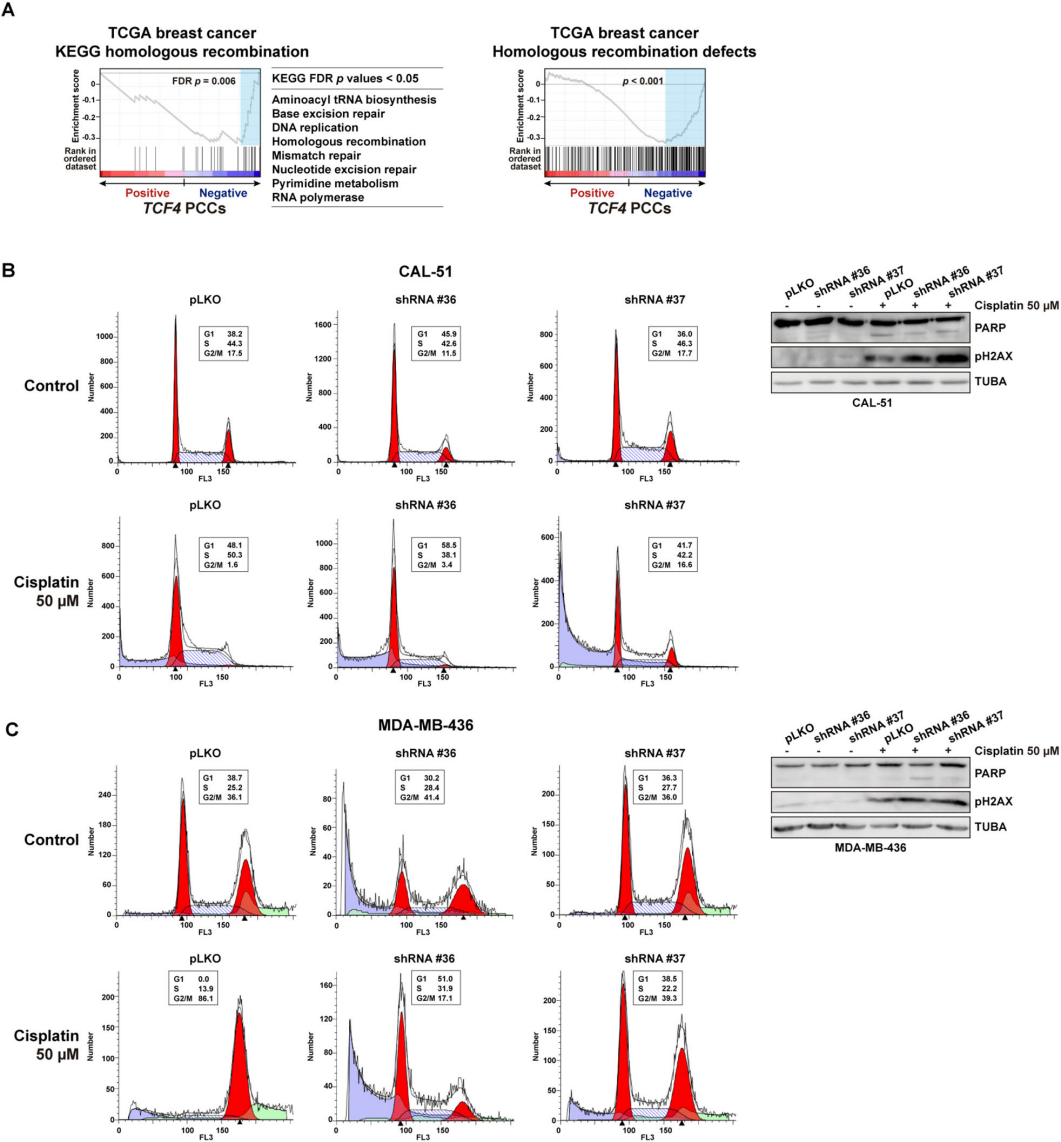
***TCF4* pathway associations and alteration of cell cycle control.** (A) GSEA results for PCCs between *TCF4* and any other gene in the TCGA breast cancer dataset. The left and middle panels show the negatively correlated (FDR <5%) pathways linked to DNA repair. Right panel shows the result of the signature of homologous recombination defects and chemoresistance. (B) Left panels show the cell cycle profiles of control (top) or cisplatin (bottom) CAL-51 cells transduced with control pLKO.1 or an shRNA targeting *TCF4* expression. The percentages of cells in the G1, S and G2/M phases are shown. Right panels so western blot results for PARP, pH2AX and TUBA (loading control) in the corresponding cell assays. (C) Cell cycle profiles and western blot results for assays of MDA-MB-436 cells.

Next, we aimed to assess the functional role of TCF4 using breast cancer cell lines. Two triple-negative cell models were selected for this study on the basis of their previously defined sensitivity to cisplatin and olaparib (Lehmann et al., 2011): CAL-51 and MDA-MB-436, the latter being a *BRCA1*-mutant model. Using two different short-hairpin RNA sequences, partial depletion of *TCF4* expression led to a significant increase in the survival of both cell lines when exposed to cisplatin or olaparib (Fig. 3B). In addition, whilst MDA-MB-436 is intrinsically resistant to fluorouracil, CAL-51 also showed resistance to this drug after partial depletion of TCF4 (Fig. 3B). Moreover, the expression profile of *TCF4* in the TCGA dataset was found to be negatively correlated with multiple pathways of DNA repair (Fig. 4A, left panels); that is, tumors with low *TCF4* expression tend to have higher expression levels of genes involved in these processes. Similarly, analysis of the signature of homologous recombination defects and chemoresistance (Peng et al., 2014) showed a negative correlation with *TCF4* expression (Fig. 4A, right panel).

To further assess the function of TCF4 in a process linked to chemoresistance, the cell cycle profiles were analyzed. Thus, TCF4 depletion led to a partial bypass of the S and G2/M cell cycle checkpoints in cisplatin-exposed CAL-51 and MDA-MB-436 cells, respectively; the population of CAL-51 cells in S phase was reduced by 8-12% (while G2/M increased by 2-15%) and the population of MDA-MB-436 cells in G2/M phase was reduced by 50-70% (Fig. 4B,C). Of note, CAL-51 and MDA-MB-436 differ in *TP53* status, the former being wild-type and therefore able to arrest in S phase (Vogelstein et al., 2000). Analysis of apoptosis and DNA damage signaling through PARP cleavage and phospho-Ser139 histone H2AX (pH2AX) levels, respectively, was inconclusive with regard to specific alterations, with the exception of a relative increase of pH2AX in TCF4-depleted CAL-51 exposed to cisplatin (Fig. 4B). Therefore, loss of TCF4 *in vitro* confers chemoresistance at least partially by perturbing cell cycle control.

### Loss of TCF4 expression post-chemotherapy

**Fig. 5.**
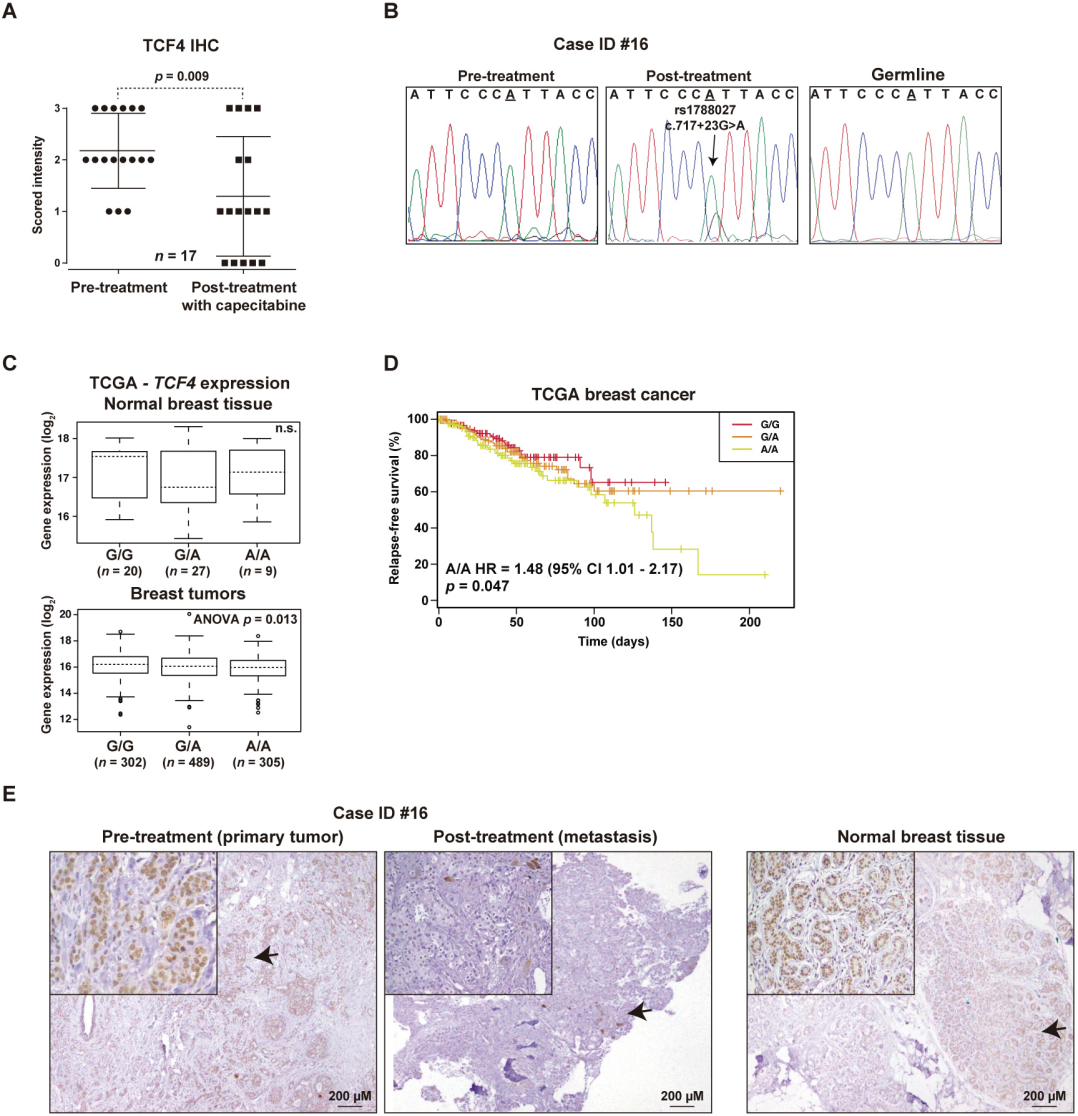
**Loss of expression and mutation of *TCF4* post-chemotherapy.** (A) Graph showing the IHC scores of paired pre- and post-treatment tumors of patients treated with fluoracil-based therapies. The Wilcoxon matched-pairs signed-rank test *P*-value is shown. (B) *De novo* intronic *TCF4* mutation in metastasis post-chemotherapy (pre-treatment and germline results are also shown). (C) Plots of eQTL evaluation between rs1788027 and *TCF4* expression in normal breast tissue [top panel; not significant (n.s.)] or tumors (bottom panel; ANOVA test *P*-value is shown). (D) Kaplan–Meier curves showing relapse rates over time for TCGA patients stratified according the genotypes of rs1788027. The HR estimation, 95% CI and log-rank test *P*-value are shown. (E) IHC results of the case showing the *de novo* intronic mutation (left panel, primary tumor; middle panel, metastasis post-treatment) and of normal breast tissue from a healthy woman (right panel). Arrows mark magnified fields shown in the insets.

To evaluate the above conclusions in a relevant clinical setting, we analyzed the expression of TCF4 by immunohistochemistry (IHC) in paired pre- and post-therapy tumor or metastasis samples from 21 breast cancer patients treated with fluoracil-containing chemotherapies. In this study, the TCF4 immunostaining scores were found to be significantly lower post-therapy (Wilcoxon matched-pairs signed-rank test *P*=0.009; Fig. 5A), further supporting the link with cancer adaptation to chemotherapy.

Next, to investigate somatic genetic alterations of *TCF4*, its coding sequence and splice sites were sequenced in five of the above post-therapy samples with cancer cell content >70%; whilst many low-frequency mutations (allele frequency <10%) might appear post-therapy, one case with relapse after 139 months revealed a *de novo* intronic mutation that may affect *TCF4* splicing regulation (c.717+23A>G; Fig. 5B and Table S7). This mutation was confirmed by Sanger sequencing in two different specimens post-therapy, but it was absent in two different pre-therapy specimens and in the germline sample (Fig. 5B). Interestingly, this genomic position is also a common germline variant, rs1788027, which may therefore have implications for the therapeutic response of other individuals. The A-allele associates with lower *TCF4* expression in different human tissues or organs [expression quantitative trait locus (eQTL) *P*-values=0.02-0.0001] (GTEx Consortium, 2013). Analysis of TCGA data confirmed that rs1788027 is an eQTL for *TCF4* in breast tumors (Fig. 5C). Intriguingly, considering the complete TCGA cohort, the cases with the inferred A/A genotype revealed a higher proportion of tumor relapses: HR=1.48, 95% CI 1.01-2.17, *P*=0.047 (Fig. 5D). The study by cancer subtypes showed similar trends and revealed significance for ER-positive cases (HR=4.72, 95% CI 1.03-21.59, *P*=0.045).

Detailed IHC evaluation of the case with a *de novo TCF4* mutation showed loss of expression of the corresponding gene product in the metastatic lesion post-chemotherapy; in fact, few cells showed positivity, but were cytoplasmic (Fig. 5E). Of note, normal breast tissue from healthy individuals shows nuclear positivity of TCF4 in the luminal-differentiated layer (Fig. 5E, right panel), which is consistent with its expression from mammary epithelial proliferation to differentiation (Itahana et al., 2008) and its negative regulatory link to DNA-binding protein inhibitor ID1 (Parrinello et al., 2001).

## DISCUSSION

Understanding the mechanisms of cancer therapeutic resistance is fundamental to improving cancer care. The main cause of cancer death is the appearance of metastases and, most frequently, metastases develop as a consequence of or in parallel with resistance to therapy. These observations are probably more relevant for TNBC. Many studies have shown significant clinical benefit of chemotherapy in the neoadjuvant, adjuvant and metastatic settings of this cancer subtype (Joensuu and Gligorov, 2012; Perez et al., 2010). Paradoxically, TNBC generally shows a better initial response to chemotherapy than other subtypes (Carey et al., 2007; Liedtke et al., 2008; Rouzier et al., 2005; von Minckwitz et al., 2012, 2014; Cortazar et al., 2014; Masuda et al., 2017). However, pathologic complete responses are only achieved in 30-40% of early-stage cases and patients who do not show this response level have 12 times the risk of death (von Minckwitz et al., 2012). Indeed, fewer than 30% of metastatic cases survive 5 years after diagnosis (Bonotto et al., 2014). In this scenario, while different therapeutic approaches are being developed and tested, there is still a need for better understanding of the genetic determinants of chemoresistance. This study contributes to this understanding by describing novel mutations and the corresponding genes possibly mediating chemoresistance.

Our results are consistent with the notion that resistance may be established through mutations in a variety of genes, which may present functional interplay but show little overlap for precise gene identities (Lønning and Knappskog, 2013). The validity of our PDX-based modeling approach is supported by: (i) observations of different proportions of deleterious mutations and functional associations, (ii) analyses of prognosis in human breast cancer data, and (iii) correlations with therapeutic sensitivity profiles in cancer cell lines. Detailed examination of gene identities reveals them to be consistent with expectations (i.e. mutations in DNA-repair-linked genes *DNTT* and *XRCC6*) but also expands on recent observations: the *de novo* gene set includes *DPYD*, whose product has been implicated in homologous recombination (Someya et al., 2012), and genetic variation has been associated with fluorouracil toxicity, which may have further implications for personalized medicine (Del Re et al., 2017). However, our study design did not assess the influence of drug holiday (i.e. intermittent treatment) on the potential reversion of the resistant phenotype or, by extension, on the role of the identified genes and/or mutations in different settings of acquisition of resistance and/or adaptation to chemotherapy.

Guided by the evidence obtained, we assessed the functional impact of a commonly mutated gene in our modeling study, *TCF4*. The corresponding gene product has been implicated in the regulation of epithelial-mesenchymal transition (Cano and Portillo, 2010) and is expressed in the progression from proliferation to differentiation during pregnancy in mammary gland development (Itahana et al., 2008). In addition, TCF4 physically interacts with the inhibitors of basic helix-loop-helix (bHLH) transcription factors ID1 and ID2 (Langlands et al., 1997). While there is no consensus as to the prognostic impact of ID2 expression in breast tumors, relatively high ID1 expression has consistently been associated with poor prognosis (Lasorella et al., 2014) and, consequently, with invasive and metastatic capacities (Fong et al., 2003). In fact, ectopic overexpression of ID1 in mammary epithelial cells promotes proliferation and invasion, and TCF4 counteracts these effects (Parrinello et al., 2001). Importantly, a *TCF4* splicing mutation with loss of heterozygosity has recently been identified in a study of therapy-naïve synchronous metastatic breast cancer (Ng et al., 2017). Thus, based on our results, metastases with alterations in this gene or protein may further appear as chemoresistant and less curable.

Cell-based assays confirmed that TCF4 loss mediates chemoresistance, and immunohistochemical study of tumors treated with chemotherapies including fluoracil showed consistent results. The identified *de novo* mutation and the corresponding IHC results in a given case further suggest that alteration of TCF4 expression mediates chemoresistance and, in turn, indicate the possible involvement of germline variants in influencing chemotherapeutic responses. Of note, the study of cisplatin response in ovarian cancer identified loss of chromosome 18q, including *TCF4*, as being associated with resistance (Bosquet et al., 2014). Nonetheless, studies of larger series with pre- and post-chemotherapy cancer samples may be warranted to decipher the precise mechanism by which altered TCF4 function is linked to chemoresistance. If confirmed, loss of TCF4 could guide the identification of biomarkers of chemoresistance, based for example on the analysis of its expression or targets. In parallel, these findings may emphasize novel therapeutic approaches by combining chemotherapy with inhibitors of epithelial-mesenchymal transition and/or ID1 function. Finally, other genes identified in the study could open up similar opportunities.

## MATERIALS AND METHODS

### PDX model

The PDX model was derived from a breast cancer patient carrying a germline *BRCA1* mutation and diagnosed at 33 years of age. At diagnosis, the patient presented a T4 ductal infiltrating triple-negative tumor with involvement of ipsilateral nodes (N2) and lung metastasis. Primary systemic chemotherapy was initiated with TAC regimen for four cycles, followed by mastectomy to prevent local complications due to extensive breast involvement. Following surgery, the patient received additional chemotherapy with the same regimen. Shortly after, brain metastases were diagnosed and the patient died 8 months post-diagnosis. The primary tumor specimen was obtained at the Institute of Oncology (ICO), Hospital Germans Trias i Pujol, Spain and mutational analysis was carried out by the Molecular Diagnostics Unit (ICO, Hospital Duran i Reynals, Spain) in compliance with the relevant standards for genetic testing and pathological determination. The patient provided written informed consent and the study was approved by the Bellvitge Institute for Biomedical Research (IDIBELL) Ethics Committee. Non-necrotic tissue pieces from the tumor were selected and placed in Dulbecco's modified Eagle medium (BioWhittaker) supplemented with 10% fetal bovine serum and penicillin/streptomycin at room temperature. Female athymic (*nu/nu*) mice (Harlan™) between 4 and 6 weeks of age were used for tumor engraftment into the mammary fat pad. Tumor growth was monitored two to three times per week, and grown PDXs were harvested, cut into small fragments and transplanted into additional animals. The IDIBELL Animal Facility meets Spanish government regulations and adheres to the Transparency Agreement on Animal Experimentation promoted by the Confederation of Scientific Societies of Spain (COSCE), with the collaboration of the European Animal Research Association (EARA). The facility has obtained accreditation by the Association for Assessment and Accreditation of Laboratory Animal Care International. The animals were housed in adequate specific-pathogen-free (SPF) conditions. Mice were housed in individually ventilated cages on a 12-h light-dark cycle at 21-23°C and 40-60% humidity, and were allowed free access to an irradiated diet and sterilized water. The study was reviewed and approved by the IDIBELL Animal Care and Use Committee.

### Drugs

Lyophilized lurbinectedin (1 mg vials; PM01183, PhamaMar, Madrid, Spain) was dissolved in 2 ml of water to a concentration of 0.5 mg/ml and, subsequently, dissolved in saline for injecting it into the mice. Cisplatin (1 mg/ml) and fluorouracil (50 mg/ml) solutions for infusion (Ferrer-Farma, Barcelona, Spain) were obtained from the Pharmacy Unit of the ICO. Olaparib was purchased dissolved in DMSO (Selleckchem, AZD2881, CAS number 763113-22-0) and then added to 10% 2-hydroxy-propyl-β-cyclodextrin/phosphate-buffered saline solution for injecting it into the mice.

### Drug-resistant PDX

After three PDX passages, tumor pieces of 2-3 mm^3^ were implanted in young female mice in order to develop paired drug-resistant models. Thus, cisplatin-, fluorouracil-, lurbinectedin- and olaparib-resistant PDXs were generated by iterative cycles of *in vivo* drug exposure, as described previously (Vidal et al., 2012). Briefly, the PDX from the third passage after initial engraftment was allowed to grow to a volume of 300-500 mm^3^ and then exposed to the corresponding chemotherapeutic regimens. The animals were intravenously (i.v.) administered with cisplatin with a dose of 3 mg/kg body weight for 3 consecutive weeks (days 0, 7 and 14; cycle #1 of treatment). Post-cisplatin tumor relapse was harvested, prepared as previously described and engrafted in additional animals. This process was repeated four times (total of five cycles of chemotherapy) by treating tumor-bearing mice with stepwise increasing doses of cisplatin: 3.5, 4 and 5 mg/kg body weight in cycle #2, #3 and #4, respectively. For the rest of the drugs analyzed, the same dose was used during the iterative cycles: (i) lurbinectedin was i.v. administered once per week for 3 consecutive weeks (days 0, 7 and 14) at 0.18 mg/kg body weight diluted in 2-hydroxyl-propyl-β-cyclodextrine; (ii) fluorouracil was administered by intraperitoneal injection (i.p.) at a dose of 50 mg/kg body weight (days 0 and 1) at week 1 and 3; and (iii) olaparib was administered daily during 21 days i.p. at a dose of 50 mg/kg body weight diluted in 10% 2-hydroxyl-propyl-β-cyclodextrine/phosphate-buffered saline. Post-treatment tumor relapses (defined as tumor volume of 500-750 mm^3^) were harvested, prepared as previously described and engrafted in additional animals.

### Exome sequencing

The National Centre for Genomic Analysis (CNAG) carried out exome sequencing by contract. Sequence capture and amplification were performed using the Agilent SureSelect Human All Exon kit (Agilent) according to the manufacturer's instructions. Paired-end sequencing was performed on a HiSeq2000 instrument (Illumina) using 76 base reads. Reads were aligned to the reference genome (GRCh37) and BAM files were generated using SAMtools (Li et al., 2009). Duplicates were removed using SAMtools and custom scripts, and single-nucleotide variant calling was performed using a combination of SAMtools and *Sidrón* algorithms, as described previously (Puente et al., 2011). For PDX-derived samples, reads were first aligned to the mouse genome (mm9), and read pairs that did not align to mouse were then aligned to the human genome following the same pipeline as above. This procedure removed murine-derived reads, which otherwise could interfere with the analysis by artificially increasing the number of variants/mutations. However, this filtering could have led to the removal of certain human genes with a very high sequence identity to mouse genome, so relevant chemoresistance-associated mutations may have been missed. Additional limitations may include poor sequence coverage in other genes. Common variants, defined as those present in dbSNP135 with a minor allele frequency >1%, were filtered out. Point mutations were called if they were covered at least 20-fold and the mutant nucleotide was present in at least two independent reads. Frameshift mutations were called if the mutant sequence was present in at least two reads regardless of the coverage of the given locus. Allele frequency (AF) was determined as the number of mutant reads divided by the number of total reads.

### Deep-targeted sequencing

The 20 PCR products from seven samples were purified using the QIAquick PCR Purification Kit (Qiagen) and sequencing libraries prepared following the manufacturer's recommendations (Illumina). The run was completed using a MiSeq Reagent Kit V2 in a MiSeq instrument with paired-end mode. Reads were mapped to the human reference genome using the Burrows–Wheeler Aligner (Li and Durbin, 2010) and variants extracted using SAMtools (Li et al., 2009). For differences relative to the reference, only high-quality bases (Phred score >30) were considered, and per-base error rate was determined from amplicon positions not affected by mutations or variation. Those positions detected in a sample at an allele frequency below the per-base error rate were considered to be below the detection limit. For small insertions and deletions, the error rate was found to be very low and total allele frequency is reported if detected (Table S5).

### Mutation classification

Mouse sequences and human germline variation were filtered out from the PDX exome data to identify somatic mutations. Mutations annotated as synonymous, intronic or untranslated, and in-frame deletion/insertions were excluded from further analyses. Prediction of the functional impact of missense mutations was performed using the SIFT (Sim et al., 2012) and Polyphen-2 (Adzhubei et al., 2013) algorithms. The mutations were classified as deleterious (including frameshift, nonsense, canonic splice sites and missense variants predicted to be deleterious by both algorithms) or non-deleterious (including the remaining missense variants).

### Mutation classification relative to chemoresistance

Somatic mutations were further classified as being linked to therapeutic resistance according to the following criteria:

#### *De* *novo*

Present in a chemotherapy-adapted PDX but completely absent from the parental PDX (i.e. no detected read at a given position/locus with at least 20-fold coverage).

#### Completely depleted

Present in the parental PDX but completely absent from a given adapted model.

#### In common

Present in both the parental and adapted PDXs, including at low frequency in either setting (minimum of one read with 20-fold coverage). A Fisher's exact test followed by FDR-based correction was used to assess whether each of the common mutations was significantly enriched or depleted. To exclude subtle changes that might not be biologically relevant, only those mutations that changed (enriched or depleted) with a percentage ≥10% were considered in further analyses.

### Mutation validation

A selection of missense changes predicted to be deleterious, and *de novo* and enriched frameshift, nonsense and splice site mutations, underwent Sanger-based sequencing validation. Primers were designed to amplify the selected genomic positions while avoiding homologous mouse sequences. To test human specificity, absence of amplification was confirmed using a mouse-only DNA sample. The PCR products were treated with ExoSAP-IT (Affymetrix) and sequenced using the BigDye Terminator v3.1 Cycle Sequencing kit (Thermo Fisher).

### Pathway enrichment analysis

The KEGG dataset of pathway annotations was downloaded from the corresponding repository (Kanehisa et al., 2017). Statistical significance of pathway enrichments was assessed using 2×2 contingency tables and Fisher's exact test. Values of *P*<0.05 after FDR-based correction were considered significant.

### Gene expression and survival analyses

For the correlation analysis between basal gene expression and the drug response across cancer cell lines, data were downloaded from the Genomics of Drug Sensitivity project (Garnett et al., 2012). This dataset included half maximal inhibitory concentration (IC_50_) values for 131 drugs that were assessed in a panel of 638 human cancer cell lines. Correlations were computed using the PCC. Pre-processed and normalized data on normal breast tissue and primary breast tumors were taken from TCGA repository (Cancer Genome Atlas Network, 2012); somatic mutation and genetic variation data was obtained following approval by the Data Access Committee (project #11689). The multivariate Cox regression survival analyses of somatic mutations (adjusted by age at diagnosis and tumor stage) included 715 cases with complete data and 67 events. The Gene Set Analysis (GSEA) (Subramanian et al., 2005) tool was run with default values for all parameters, using curated (C2) or KEGG gene sets, and a signature of homologous recombination defects and chemoresistance (Peng et al., 2014), and the *t*-statistic or PCC values as rank metric.

### Cell culture and antibodies

The CAL-51 and MDA-MB-436 cell lines were cultured in standard conditions. The cell lines were tested and found to be authentic, and *Mycoplasma* contamination was tested periodically. Cellular viability was evaluated using methylthiazol tetrazolium (MTT)-based assays (Sigma-Aldrich). TCF4 expression was assessed in western blots using a rabbit polyclonal antibody: Antibodypedia catalog number ABIN184295. The expression depletion assays used MISSION shRNAs (Sigma-Aldrich) catalog number TRCN0000015036 and TRCN0000015037. The lentiviral packaging, envelope and plasmids psPAX2, pMD2.G and non-hairpin-pLKO.1 were purchased from Addgene. Additional antibodies used in this study were anti-phospho-Ser139 histone H2A.X (2577, Cell Signaling), anti-PARP (551025, BD Pharmingen) and anti-tubulin-α (ab44928, Abcam). Cell cycle analysis was performed following a standard protocol of DNA staining with propidium iodide. Flow cytometry analysis was performed using FACS Gallios (BD Biosciences) and the ModFit software package.

### Tumors treated with chemotherapy

The patient cohort was diagnosed between 1998 and 2013 at the ICO (Hospital Duran i Reynals, IDIBELL). All patients were treated with a chemotherapy regimen based on CMF (cyclophosphamide, methotrexate and fluorouracil) or capecitabine (a pro-drug converted into fluorouracil), and all patients provided biopsies before treatment and following cancer progression. Twenty-one cases were included in the study, with a median age of 49 years (26-71). Eighteen cases had luminal-like tumors, one case had a triple-negative tumor and two cases had HER2-positive tumors. All patients received treatment with chemotherapy, one as neoadjuvant setting, 16 as adjuvant therapy after surgery and four as a first line of metastatic treatment. The median time to progression was 70 months (7-172). The patients provided informed consent and the study was approved by the IDIBELL Ethics Committee.

### Targeted sequencing

Targeted sequencing of *TCF4* was performed by the Quantitative Genomic Laboratories, Ltd (qGenomics). Briefly, DNA from paraffin-embedded tumors (pre- and post-chemotherapy paired samples) was extracted following the FFPE-DNA protocol on a Maxwell robot (Promega), according to the manufacturer's instructions. Illumina-compatible libraries were generated from the extracted DNA with the KAPA HTP library preparation kit (Roche). Prepared libraries were pooled together and hybridized against oligonucleotide baits (SeqCap EZ choice, Roche-NimbleGen) designed to cover the coding and non-coding regions of *TCF4*. Enriched pools were paired-end sequenced (2×150 base pairs) on a NextSeq500 platform (Illumina). Reads were aligned to the human reference genome with BWA (Li and Durbin, 2010) algorithms, and variant calling, annotation and filtering was performed by using GATK (McKenna et al., 2010), ANNOVAR and in-house-developed scripts (available upon request).

### Immunohistochemistry

Detection of TCF4 expression in paraffin-embedded tumor samples was performed using standard immunohistochemical assays with antigen retrieval achieved in citrate-based buffer. The antibody (H00006925-M03, Abnova) was used at a dilution of 1:50. The immunohistochemical scores were defined blindly with respect to patient status and tumor setting by two independent observers.

## Supplementary Material

Supplementary information
