# Supplementary material for: Tumor xenograft modeling identifies an association between TCF4 loss and breast cancer chemoresistance
Source: Dis Model Mech. 2018 May 18;11(5):dmm032292. doi: 10.1242/dmm.032292 (PMC5992609; doi:10.1242/dmm.032292)
Supplement: Supplementary information [file dmm-11-032292-s1.pdf]

Supplementary Figures

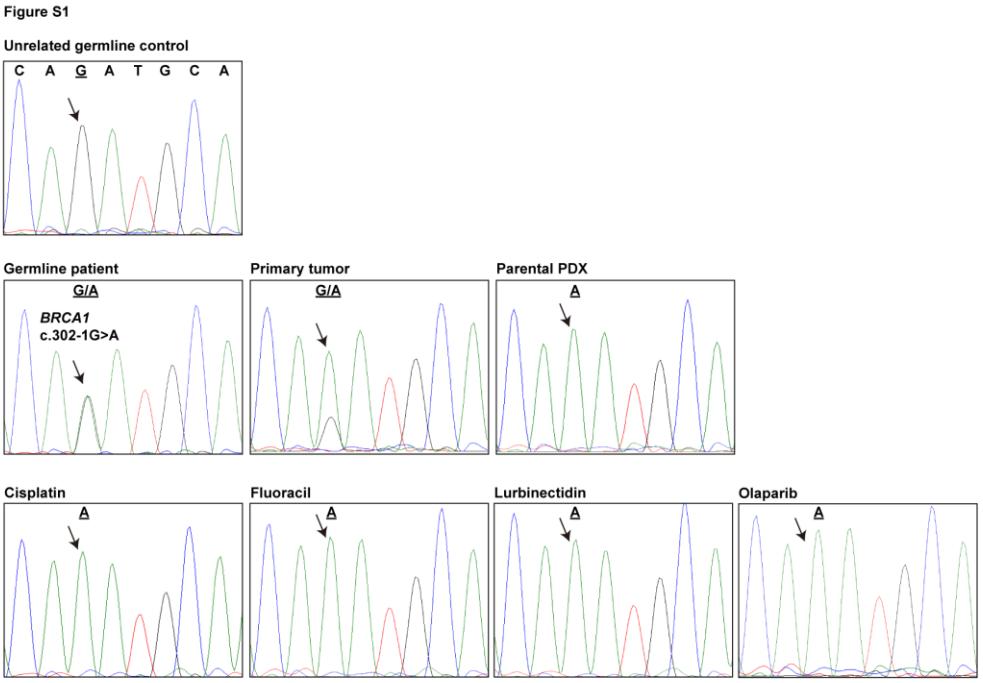

Fig S1. Results from the analysis of the *BRCA1* germline mutation in an unrelated individual, patient samples and derived PDXs.

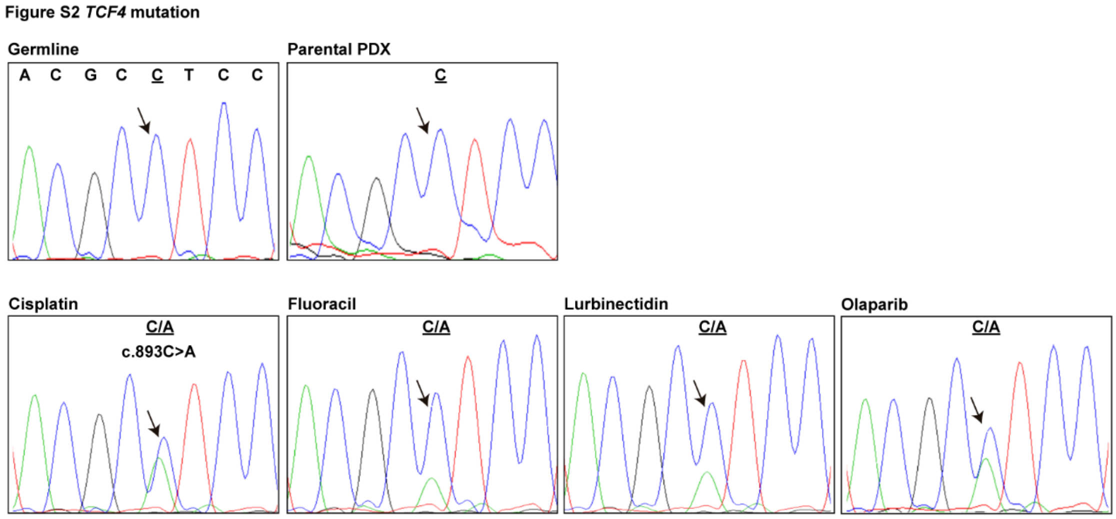

Fig S2. Results from the analysis of the *TCF4* mutation in the germline and derived PDXs.

Supplementary Tables

Table S1. Mutations identified *de novo*.

Click here to Download Table S1

Table S2. Mutations depleted.

Click here to Download Table S2

Table S3. Mutations significantly enriched or depleted.

Click here to Download Table S3

Table S4. Mutations assayed for validation.

Click here to Download Table S4

Table S5. Results from deep-targeted sequencing of 20 somatic mutations.

Click here to Download Table S5

Table S6. Deleterious mutations in the TCGA dataset (*de novo* gene set).

Click here to Download Table S6

Table S7. Results of targeted *TCF4* sequencing in five samples post-treatment.

Click here to Download Table S7
